# Queen Charlotte's Nurses' Home

**Published:** 1899-07-22

**Authors:** 


### The Institutional Workshop.

### QUEEN CHARLOTTE'S NURSES' HOME.

The newly-opened nurses' home which has just been erected for the nurses and medical students attached to Queen Charlotte's Hospital is a compact and convenient building. It affords accommodation for 60 pupil nurses, 10 staff, and domestics. It also possesses a sick ward large enough for the reception of two patients. A dining-room, a sitting-room, and kitchens, &c., are placed in the basement. The home sisters' sitting-room, that of the superintendent of the out-patient department, and the bedrooms of the nurses engaged on district work are found on the ground floor. The four upper floors are devoted to bedrooms, one being assigned to each nurse.

The rooms are high and fairly large; they are prettily papered, and the woodwork is planed and varnished. The floors are stained, and the furnishing adequate and very nice. It is light in tone and matches the woodwork. The oak staircase is especially pretty, and close by is a luggage lift. Under the same roof, but quite separate, are the apartments reserved for the medical students, the only communication between the two being the serving hatchway in the kitchen—the one kitchen serving both residences. The building is from the plans of the honorary architect, Mr. F. W. Hunt, who also superintended the building. The contract was executed in time, in spite of difficulties owing to the plasterers' strike, by Messrs. G. H. and A. Bywaters and Sons.

